# The role of universities' sustainability, teachers' wellbeing, and attitudes toward e-learning during COVID-19

**DOI:** 10.3389/fpubh.2022.981593

**Published:** 2022-07-27

**Authors:** Melinda Timea Fülöp, Teodora Odett Breaz, Xiaofei He, Constantin Aurelian Ionescu, George Silviu Cordoş, Sorina Geanina Stanescu

**Affiliations:** ^1^Faculty of Economics and Business Administration, Babeş-Bolyai University, Cluj Napoca, Romania; ^2^Faculty of Economic Sciences, 1 Decembrie 1918 University, Alba Iulia, Romania; ^3^Lucian Blaga University, Sibiu, Romania; ^4^Hangzhou College of Commerce, Zhejiang Gongshang University, Hangzhou, China; ^5^Institute of Multidisciplinary Research for Science and Technology, Valahia University of Targoviste, Targoviste, Romania; ^6^Faculty of Economics, Hyperion University Bucharest, Bucharest, Romania; ^7^Transylvania Business School, Babeş-Bolyai University, Cluj Napoca, Romania

**Keywords:** sustainability, e-learning, wellbeing, TAM, attitudes

## Abstract

In recent years, universities worldwide have experienced rapid changes with an immense impact, which have been influenced by technological progress and the social trends of digitalization. Like all other revolutionary changes, digital transformation involves intense adjustment/readjustment. University sustainability must be the active concern of all higher education institutions. Thus, the present research aims to analyse teachers' acceptance of new technologies and the impact on their wellbeing and university sustainability. The main objective was to analyse the acceptance of technology in special the e-learning opportunities and the wellbeing of teacher in an emergent country like Romania. To achieve our goal, we created a questionnaire based on the literature, and with the help of the technology acceptance model, we tested our hypotheses. The results indicate several discontents on the part of teachers concerning adapting to new technologies and even a personal discomfort in adapting to these new technologies. Thus, we can note that wellbeing significantly influences job satisfaction and teachers' involvement in sustainable development.

## Introduction

Today's working world has become very complex in many areas: processes are becoming more concentrated and often make it difficult for us to “switch off” in the truest sense of the word, with consequences for our wellbeing. Changing the world may be difficult, but individuals can do something for themselves and their mental health.

The concept of sustainability is more present than ever in our society and not just because of the growing presence of the *Fridays for Future* movement. Originally initiated by schoolchildren, the almost weekly demonstrations have also gained popularity among the general public. Due to the recent heated debate on climate change, many other sustainability issues have arisen in society and politics, which are also widely discussed. Society increasingly demands that companies take responsibility in this area (1).

However, not only do consumer goods manufacturers need to answer their customers' questions about sustainability, but service providers are also being monitored more and more closely. Sustainable corporate management is becoming increasingly important, and the relevance of corporate social responsibility is growing. This shift does not stop at educational institutions. Within universities, the theme of sustainability can be divided into two main aspects, namely, the educational mission of the university, and the institution itself, through its actions. As a knowledge broker, the company also expects a specific commitment to sustainability and, in general, environmentally conscious behavior from an educational institution. Universities fulfill a particular function as a model in the content it delivers. Nevertheless, in addition to social assessment, it is beneficial to consider and include sustainable issues in the management of a university. On the one hand, the economical use of resources can lead to cost advantages; on the other hand, a sustainably managed university operation can improve the university's reputation.

Sustainability can be exanimated with a macro method connected to the general economic structure, and a micro method, placing the examination on precise personnel. With regards to a commercial level, corporate sustainability can be definite as consulting the requests of a company's direct and indirect investors, not containing its aptitude to reply to the requirements of forthcoming investors (2–6). Corporate sustainability can be articulated in diverse ways, as businesses must produce and preserve the interrelated economic, social, and environmental resources, particularly if sustainability is anticipated in the long run (7, 8). In addition, Corporate Social Responsibility (CSR) has established important consideration in academic and professional discussions: scientists consider that businesses should recover the social and environmental effects of their activities, but their efforts would be directly related to the commercial approach of a business (9, 10).

We believe this paper contributes to the literature in being a good guide in accepting new technologies in academia, at least for the following reasons. First, this paper analyses the literature on sustainability and digitalisation in the university field. The novelty brought by the research aims at the impact of this process in the Romanian academic environment. So far, various studies have been carried out on the model of accepting technology in the university environment in different countries. However, we have not found such a study for Romania, so we deemed it worthwhile to analyse the situation in an emerging country like Romania. To achieve the objectives, we started with the literature analysis, followed by the presentation of the fundamental theories on education in the context of e-learning and the acceptance of technology. The theoretical component is followed by a practical part in which we present our study's results based on the technology acceptance model. We end the paper with a series of conclusions and research perspectives.

## Analysis of literature on the role of sustainability in the university environment

Research on education for sustainable development in current research is grounded on the tradition of broader investigation with regards to curriculum modification. In the last decade, there have been an increasing number of papers on curriculum modification procedures in universities for sustainable change (11–13).

**Figure 1:**
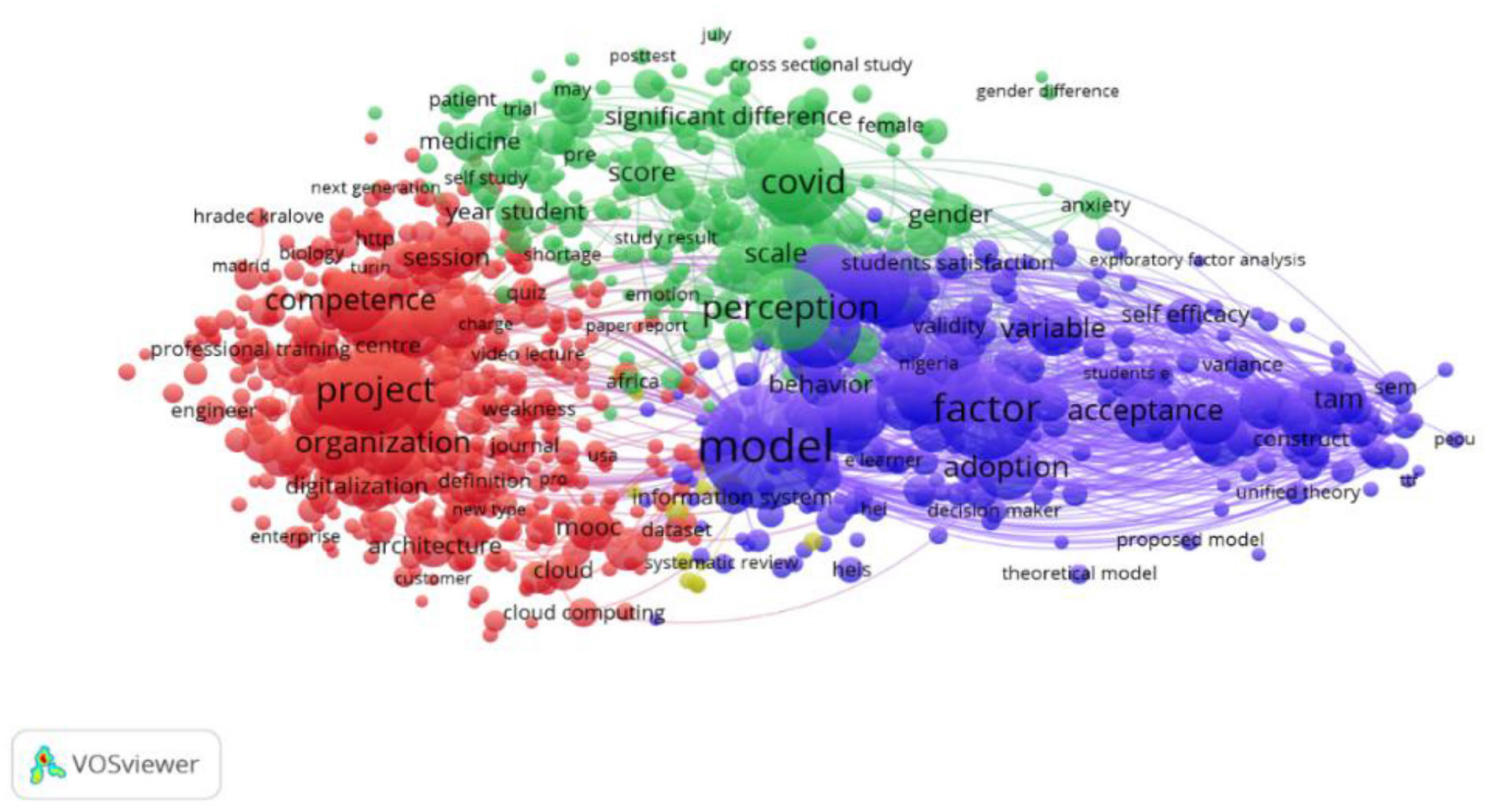
Academic e-learning cluster.

“Keyword analysis on e-learning” (see Figure 1) showed a more detailed and interconnected model between the impact of the pandemic, digitisation, and e-learning. Therefore, it is clear that the pandemic significantly impacted the digitisation and transition to an e-learning type of learning.

The green cluster aims at the effect of the pandemic on higher education, directly connected to the part of e-learning represented by the purple cluster. The red cluster represents digitisation and the digitisation process in the university environment.

E-learning and blended learning are essential elements of contemporary university teaching. The study models offered are as diverse as the students. With the help of innovative teaching concepts, students' learning styles can be more easily approached on the one hand, and on the other hand, we can discuss the different learning materials. Universities worldwide support students and faculty in creating and using digital courses, especially during this pandemic (14, 15).

The digitisation of higher education has recently gained widespread momentum. Although e-learning elements have been introduced since early 1990 s, there is a discussion regarding virtual universities and online studies. In addition, workshops were organized to address new media challenges (16).

In this situation, existing university e-learning strategies are checked for compatibility with current strategic guidelines. At the same time, the development of a comprehensive digitisation strategy that complements or replaces an e-learning strategy is often discussed. After a series of failures at the beginning of the millennium, awareness prevailed that e-learning in universities should not only be used as product innovation in teaching, but as a process innovation in the organization of the university. Therefore, for a long-term anchoring of e-learning, it seemed necessary not only conceiving educational media concepts, but also viable strategic guidelines for university management, developed as much as possible in a participatory process (16, 17).

Particular attention is paid in the literature to e-learning strategies and a digitisation strategy that goes beyond teaching and study. We elaborated on the advantages and disadvantages of focusing on digitalisation and sustainability of higher education with the help of the following dimensions: organization, economy, culture, and the process of change/leadership.

In a nutshell, the following conclusions can be drawn for keyword analysis. First, it was noticed that there were differences depending on terminology, referred to the aggregation level and the focal points in the content. On the other hand, the topics and considerations at the university level were found in all terms. Especially at the individual level, the ability to transfer, get involved and the competence in sustainability related to the topic of education. The different terms have all been viewed in the context of sustainable development and digitisation. They have shown intersections in R&D, technology, and information management, especially during the pandemic. On closer inspection of the implications of the pandemic, we can see a close link between digitisation and the sustainability of universities.

### From fundamental theories of learning to didactics in the e-learning context

Behaviorism, cognitivism, and (socio-) constructivism have developed before the digital age (18, 19). Can these “pre-digital” learning theories describe or shape learning in the digital age? (20). If one asks the Canadian media education specialist Siemens, the answer is clearly “No!” George Siemens does not leave “no,” but with “connectivism,” he offers a model that claims to be a theory of learning for the digital age. Siemens' initial view is that the learning opportunities resulting from the Internet cannot be processed by the “classical theories of learning,” such as behaviorism, cognitivism, and (socio-) constructivism. Therefore, it is essential to express a theory of learning for the digital age: Behaviorism, cognitivism and constructivism are the three general theories of learning, most often used in creating learning environments (21–23).

Reversed classroom approaches also provide virtual knowledge transfer, for example, video lectures to prepare or Blogs, wikis and social networks allow for social and collaborative learning. Blended learning, on the other hand, is based on integrating digital content into face-to-face formats, i.e., a link between the online and offline phases (24, 25). This accomplishes a variety of classroom methods—and electronic tools complement the social aspect of personal communication. Optional, selective enrichment of classroom teaching, for example, through a PowerPoint presentation, must be distinguished from this. Electronic assessments and exams are available for preparing digital exams with fast feedback and actual exams and assessments. Pioneering projects promise the use of artificial intelligence (AI), augmented reality (AR) and the integration of playful elements (gamification) or 360-degree videos.

Vocabulary learning, conversation exercises, exam preparation, technical discussions, or direct access to materials: Digital learning opportunities, also known as “Using Information and Communication Technology (ICT) to Learn,” are used as a variety of learning tools. They contrast with the “learning to use ICT” approach, in which digital media manipulation is learned as an end in itself, for example, using an Office program. Stationary computers or mobile devices, which can be used in many different ways, are indispensable for digital learning (26, 27).

A look at teaching practice shows that many formats and forms of application of digital higher education that can be found in universities. Online courses largely complement the classic face-to-face teaching. For example, students prepare for video seminars, complete self-learning programs during the event, or create group presentations using digital media. In addition, the university promotes a full range of blended learning formats, meaning that teaching and learning videos, virtual labs and interactive learning platforms are part of students' daily lives.

The didactic problem is also closely related to the reflections on the learning theory. In other words, reflections on the theory of learning make it possible to lay the foundations of didactics. In principle, teaching can be defined as a method. As a method, didactics can be understood as a regulated procedure for developing teaching/learning scenarios and as a moderation strategy in teaching/learning contexts (28). Didactics as a method is preceded by “methodology” or reflection: what is teaching and learning and how and why teaching and learning should take place.

Learning theories can be understood as teaching methodology. By linking didactics to learning theory, it is possible to provide a scientific basis for teaching models.

Integrating new media into teaching takes advantage of the almost explosive increase in internet users among students. The flexibility of studies with the help of new media has a possible added value for these groups more than their conventional counterparts. However, this can only work if target groups also have adequate access to e-learning opportunities.

As a rule, first-year students already have several previous experiences with new media. The individual acquisition of new media in adolescence is more leisure-oriented and hedonistic. Computers and the Internet are used primarily to provide entertainment (music, movies, games), get information about leisure activities, and communicate with other young people. They are primarily more straightforward instrumental skills in dealing with standard applications that are acquired in the context of media socialization.

However, the ability to reflect on the media and it usage, and creative-active usage is less pronounced. Thanks to previous experience, most students quickly find their way into campus information systems and learning platforms. When it comes to downloading course materials or e-mail communication, most students have no problems—especially if the browser or the e-mail is familiar.

In addition to this knowledge and skills, motivational requirements are also necessary. As has been repeatedly shown in various studies, the increased use of new media is accompanied by expectations. Students expect their teachers to be available by e-mail or instant messaging chat and support courses with electronic materials.

Integrating new media into teaching is already based on the individual requirements of students. However, it also creates new situations where one can practice dealing with new environments and learn new applications. Therefore, the use of new media in the context of teaching is in no way reduced to the role of mediator. On the contrary, the fact that the use of new media in teaching and learning contexts interacts with the aims, content and methods is always emphasized by media didactics (29–31).

The main frame of reference for integrative media teaching is all the communication contexts. Communication using new media—inside and outside the courses—is part of this practice (32). Using the media is not an end, but its use is very context-dependent. Before students can access e-learning offerings, they must first learn how to operate the learning management system. What is this benefit, mainly if two or three learning platforms are used in a course? However, if students of educational sciences already use a learning management system during their studies, e.g., Moodle, which they will use later on, it can be an advantage for them. For the innovation process to be put into practice in the long term, obstacles to applying new teaching and learning scenarios must be removed.

Therefore, at this time, in the field of teachers' training, there is a plea for media priority and media scenarios that can be used in pedagogical areas of action. The consequences of integrative media teaching on a university's media development plan are obvious. Different areas of competence are already relevant throughout the study. Based on students' future action areas, other computer-supported communication and interaction processes are given prominence in educational studies (33, 34).

### The role and impact of accepting technology on wellbeing

“Acceptance” explains a behavior or attitude depending on an acceptance object. As an object of acceptance, it means technical, organizational, institutional, and social changes or innovation. In this sense, acceptance is the appropriation of something offered, available, or suggested. The different types of use require the definition of the concept of acceptance for the specific used field, so we intend to define and present an empirical study on the acceptance of technology, more precisely, the acceptance of the e-learning system (35).

The first debates on the concept of acceptance took place in the mid-1970 s. They followed the social discussion about the innovative communication technologies used to permanently change the organizational processes in companies. At the time, acceptance research was less interested in the social consequences of these new technologies. Instead, it was primarily concerned with business issues, including sales market screening, economic risk assessment, or potential analysis to prevent poor investment (36). It was not until the late 1970 s and early 1980 s that research focused on economic and social acceptance issues due to the accelerated development of technical devices and their rapid penetration into almost every area of life. This period can be described as an era characterized by an increase in technology that also affected private households (37, 38).

A significant incentive for the genesis of acceptance research based on social sciences came from the assumption of a model of the hostile attitude of the population toward new technologies. This debate has been stimulated by public opinion polls with partially unconfirmed findings, either because of a supposedly declining number of students interested in technical fields of study or because of the critical opinion of the population on the expansion of nuclear technology. However, the differentiated climate of opinion in the context of public controversies about old and new technologies has been misinterpreted as a measure of a negative attitude of the population toward new technical innovations. As a result, the description of the situation by the various stakeholders turned into a new statement in which different approaches found traction simultaneously, which is understood by difficulties of acceptance and what possibilities exist to solve them (39, 40).

However, the concept of acceptance should not be equated exclusively with the acceptance of technology. Although most publications are technology-related, the construct is discussed in almost all research areas. This broad, multidisciplinary interest in issues related to acceptance, even if not related to technology, is mainly due to the discussion on the general phenomena of social acceptance.

Acceptance is not a term derived from science but a term of everyday language. The word is used primarily in social discourse by politicians, economists, and advertisers in the form of acceptance predictions and has made an actual career use in this form. Since the 1980 s, the term has become a buzzword for advertising and the linguistic repertoire of various stakeholders.

A presentation of the definitive use of acceptance in the context of scientific questions leads to the realization that there is no uniform definition of the term here either. At the beginning of the research effort, acceptance was primarily understood as an attitude that applied to certain forms of opinion and behavior and was mixed with terms such as attitude, acceptability, or adoption. For example, Alexandre et al. understand acceptance as “an attitude of larger social groups toward individual technologies that can be determined at a certain point in time and is expressed in certain forms of opinion and behavior” (41). Similarly, but in more detail, Hilbig defines acceptance as “a more or less affirmative attitude of an individual or group toward an object, subject, or other matter” (42).

Consequently, Anstadt et al. define acceptance as an expression of a user's positive attitude toward technology, expressed in the desire to implement and use it in a specific situation (43). According to Gunasinghe et al. acceptance “contradicts the rejection term and describes the positive acceptance of an innovation by the user” (44). The use of innovation can take place on several levels of acceptance (levels of use). For example, a purely passive use would indicate a relatively low level of acceptance. In contrast, a high level of use can be depicted if a user uses innovation in various ways, i.e., beyond the expected use.

For this study, a specific definition of acceptance reflects current research findings on acceptance.

Moreover, to be happy, we must feel good about our everyday work. Above all, due to the restrictions of the pandemic and changed conditions in the home office, wellbeing is becoming increasingly important (45). When we are overly stressed, alarm bells go off in the body, and we feel unfocused and exhausted. To get out of this hamster wheel, we must care for ourselves and our bodies (46). We are aware that we perform better and feel better when we are balanced, but are companies mindful of their duty to support employees to feel good?

Today's working world is changing swiftly, and the demands on employees and managers are increasing—directly affecting wellbeing and health. Stress, the ever-increasing flood of information and work intensification quickly leads to a lack of productivity, loss of concentration and health-related days off, if there is no prevention (47, 48).

Digital technologies are increasingly penetrating our daily routines, transforming how we work, spend our free time, and interact with each other. However, while supporters of spreading these technologies expect positive effects for individuals and society, opponents fear risks such as information overload, dependencies, and loss of privacy. Given these controversies, our goal is to explore the long-term individual and societal consequences of using digital technologies (49).

In the literature, there is debate on whether digitisation's effects on individuals' lives should be assessed as predominantly positive or negative. However, with a view to future business areas, it is essential to remember that it will continue to be about fulfilling people's basic psychological needs, both online and offline. Digital tools can help meet needs in new ways, but they must be designed to do that. A fully digital approach cannot make something successful or suitable for people (50).

While e-learning has many benefits, it also comes with some challenges. Some of these challenges can even affect our wellbeing. The e-learning environment offers many advantages, such as ease of use, flexibility, and accessibility. However, while online learning offers many benefits, it also comes with challenges. These challenges can affect our wellbeing (51). The WHO defines wellbeing as a “state of complete physical, mental and social wellbeing and not merely the absence of disease or infirmity.” The top three challenges affecting learning progress and wellbeing during the e-learning process are: isolation, computer skills and setting priorities (52–56).

- *Isolation:* While self-paced learning has significant benefits, the online environment can often feel lonely. The feeling of community, in an online context, is a complicated subject, especially when completing tasks and working through modules alone. While the traditional classroom offers face-to-face encounters, conversation, and socializing opportunities, we know these types of connections are rare or impossible online (52, 55).
- *Computer skills:* With rapidly changing technologies, it can be challenging to keep up with the latest computer functions and features, especially for online platforms, as new software and other media are constantly coming onto the market. The flood of new technologies and the steep learning curves can affect our wellbeing. One may feel overwhelmed by the sheer volume of new information and skills needed to process the provided information (53, 54).
- *Setting priorities*: Online learners are usually not just learners. It can be challenging to prioritize deadlines, assignments, and examinations, especially when everything is necessary and simultaneous. Effective time management is the best way to solve this problem (56).

It is essential to realize that we must take care of our wellbeing amid chaos. Even if it takes longer to reach our goals, it is not worth sacrificing our wellbeing.

## Research methodology

A questionnaire was designed based on the literature and subsequently sent to teachers to obtain an x-ray of the status and challenges they face in adopting e-learning (Table 1).

**Table 1:** Items selection based on the literature.

1	Perceived ease of use	(57–68)
2	Perceived usefulness	(57–61, 63–73)
3	Ability to use	(57, 59, 62, 65, 66, 74–76)
4	Attitude toward use	(57, 67)
5	Satisfaction and personal development	(57, 63, 64, 68, 73, 77)
6	Behavioral intent to use	(57, 59, 61, 63, 65–68, 73, 78–85)
7	Course content and design	(65, 68, 86–88)
8	Instructor contribution	(57, 65, 89, 90)
9	Actual use	(66, 91)
10	Previous experience in e-learning	(57, 65, 67, 92, 93)
11	The quality of the e-learning system	(67, 94–96)
12	Academic performance	(64, 68, 97–100)

Specifically, the questions are about perceived usefulness and perceived ease of use. The first section deals with the personal information of faculty members that reflects their field and experience. The second section focuses on e-learning levels of use.

The 36 items include respondents' perceptions and barriers to learning resource availability, material comprehension, learning attitudes, ease of access, delivery methods, and interaction patterns. In this study, respondents' perceptions were obtained from the learning they experienced in terms of models of interaction with lecturers, interactions with other students, the availability of support facilities, including Internet networks, and the availability of teaching materials in the e-learning system. In a structured way, this perception implies indicators of perceived use and ease of use. The answers to the questions on the technology acceptance model and the items of the subjective norm were recorded using a five-point Likert scale, corresponding to the original questions: 1 = “total disagreement” to 5 = “total agreement”.

**Figure 2:**
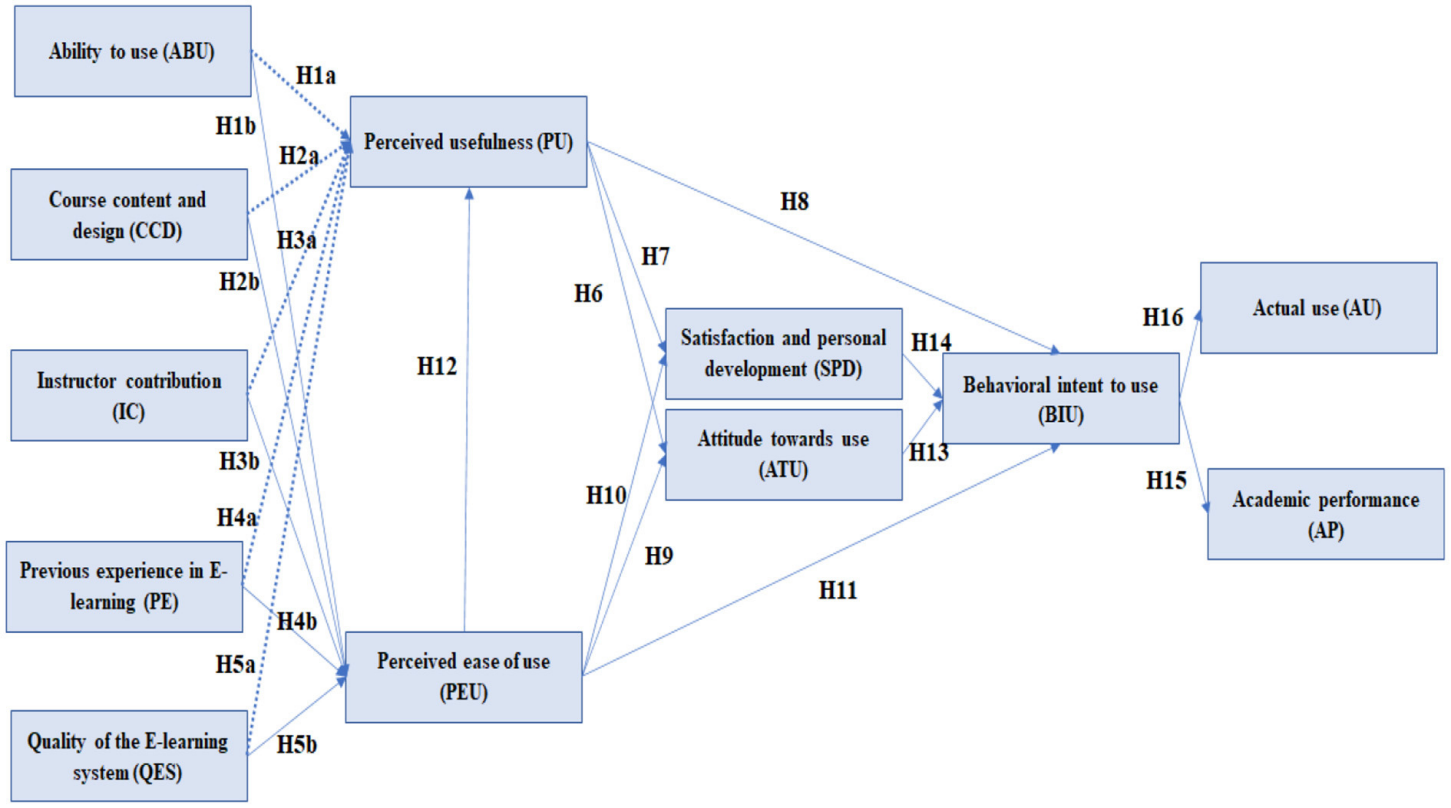
Hypothesis development.

Based on the technology acceptance model by Davis (101), we propose the following model and hypothesis to be tested (Figure 2).

The data was collected using a survey conducted using the CAWI (Computer Assisted Web Interview) technique. A link to the electronic questionnaires was distributed to teachers and individual students at Romanian universities through the university's e-mail system. These were distributed between January and February 2022. A total of 243 completed questionnaires were received from teachers.

## Results and discussion

Below we present the results obtained on the 12 elements and the 36 related items to have an x-ray on teachers' perceptions regarding the acceptance and use of the e-learning system.

Skills development is increasingly recognized as an essential condition for the sustainable anchoring of new forms of learning and media in the university. It initially refers to teachers' knowledge, skills and attitudes toward the development, introduction, and use of innovative forms of e-learning in teaching. In addition, skills development also includes an institutional level; it also affects the ability of an organization to provide specific quality services. For this reason, many universities can increase efforts to motivate teachers to innovate in e-learning and reorganize or set up processes and structures of support facilities to build this competence at the individual and organizational levels. Computer skills are only a tiny part of “e-learning skills.” The demographical characteristics of the sample are presented in Table 2.

**Table 2:** Demographic results regarding the teachers involved in the study.

	**No. answer**	**Percentage**
**Category of teachers**
Assistant	34	13.99%
Lecturer	100	41.15%
Assistant professor	75	30.86%
Professor	34	13.99%
Total	243	100%
Age group
21–30 years	25	10.29%
31–40 years	48	19.75%
41–50 years	85	34.98%
51–60 years	66	27.16%
Over 60 years	19	7.82%
Total	243	100%
**Work years (experience)**
1–5 years	36	14.81%
5–10 years	17	7.00%
10–15 years	28	11.52%
15–25 years	88	36.21%
Over 25 years	74	30.45%
Total	243	100%
**University type**
Public	224	92.18%
Private	19	7.82%
Total	243	100%

As in the case of the first and second models, we started with verifying and validating the data. Therefore, the first test applied was the validity test which indicates a value of 0.915 with a sig. 0.000, indicating that the sample is adequate (Table 3).

**Table 3:** KMO & bartlett test.

Kaiser-meyer-olkin measure of sampling adequacy		0.915
Bartlett's test of sphericity	Approx. Chi-square	1,977.877
	df	66
	Sig.	0.000

For better assurance of the suitability of the sample, we have resorted to an additional reliability test which also indicates that the sample is adequate with a Cronbach's value larger than 0.9, which indicates that the sample is, again, adequate (Table 4).

**Table 4:** Reliability test.

**Cronbach's alpha**	**Cronbach's alpha based**	**N of Items**
	**on standardized items**	
0.919	0.919	12

In order to highlight detail on factors related to each element, an analysis of the factor load was performed; respectively, we analyzed the elements' reliability, as see Table 5. All the values fall within the recommended range.

**Table 5:** Factor load and item reliability.

**Elements**	**Factor loading internal**	**Composite factor reliability** ≥**0.70**	**Convergent validity average variance extracted** ≥**0.50**
Ability to use	0.900	0.896	0.767
	0.898		
	0.770		
Course content and design	0.877	0.856	0.697
	0.889		
	0.866		
Instructor contribution	0.866	0.798	0.736
	0.898		
	0.768		
Previous experience in e-learning	0.867	0.891	0.723
	0.856		
	0.808		
The quality of the e-learning system	0.778	0.834	0.726
	0.815		
	0.903		
Perceived usefulness	0.908	0.798	0.687
	0.898		
	0.914		
Perceived ease of use	0.879	0.804	0.713
	0.799		
	0.817		
Satisfaction and personal development	0.774	0.815	0.768
	0.813		
	0.865		
Attitude toward use	0.829	0.874	0.812
	0.912		
	0.897		
Behavioral intent to use	0.829	0.813	0.674
	0.867		
	0.638		
Actual use	0.884	0.822	0.874
	0.816		
	0.874		
Academic performance	0.914	0.897	0.830
	0.902		
	0.916		

The correlation matrix of the data set is shown in Table 6. Correlations larger than 0.3 were statistically significant at 0.01.

**Table 6:** The correlation matrix.

**Elements**	**1**	**2**	**3**	**4**	**5**	**6**	**7**	**8**	**9**	**10**	**11**	**12**
Ability to use	1											
Course content and design	0.571	1										
Instructor contribution	0.611	0.639	1									
Previous experience in e-learning	0.193	0.375	0.354	1								
The quality of the e-learning system	0.459	0.477	0.603	0.307	1							
Perceived usefulness	0.572	0.721	0.501	0.381	0.384	1						
Perceived ease of use	0.639	0.582	0.498	0.371	0.355	0.691	1					
Satisfaction and personal development	0.512	0.779	0.487	0.314	0.404	0.764	0.594	1				
Attitude toward use	0.535	0.629	0.496	0.253	0.402	0.755	0.579	0.656	1			
Behavioral intent to use	0.533	0.745	0.484	0.347	0.369	0.792	0.591	0.855	0.730	1		
Actual use	0.307	0.396	0.381	0.351	0.334	0.326	0.381	0.337	0.352	0.365	1	
Academic performance	0.481	0.683	0.519	0.343	0.580	0.649	0.507	0.665	0.523	0.637	0.311	1

Finally, we present the reliability results based on the Cronbach's index, for which a value larger than 0.7 is recommended to be considered adequate. In our case, all values are above 0.9 (Table 7).

**Table 7:** Reliability of elements based on Cronbach's alpha.

**Elements**	**Media**	**Cronbach's alpha**
Ability to use	3.96	0.913
Course content and design	3.63	0.905
Instructor contribution	3.79	0.912
Previous experience in e-learning	3.30	0.927
The quality of the e-learning system	4.07	0.917
Perceived usefulness	3.61	0.906
Perceived ease of use	3.95	0.912
Satisfaction and personal development	3.41	0.905
Attitude toward use	4.19	0.910
Behavioral intent to use	3.43	0.906
Actual use	4.33	0.924
Academic performance	3.81	0.910

In order to determine the fit and suitability of the model, the analysis was performed using IBM SPSS AMOS 26 Graphics. Following the analysis of the model fit indices, it is observed that it is suitable, as shown in Table 8.

**Table 8:** Fit indices.

**Indicator**	**Recommended values**	**Values obtained**
Chi square	<3.00	1.943
GFI	>0.90	0.915
AGFI	>0.80	0.840
NFI	>0.80	0.913
CFI	>0.90	0.967
RMSEA	<0.10	0.071

It was necessary to perform preliminary tests to ensure that the elements used in the model are validated and reliable and meet the matching criteria to validate the formulated hypotheses. Thus, in Table 9, we present the results of the estimates based on the path analysis (Path coefficient) to validate or reject the previously formulated hypotheses. Ten of 21 hypotheses were rejected due to a higher value for P of 0.001, indicating no or insignificant influence between variables (Table 9).

**Table 9:** Results of the study.

	**Path coefficient**	***P***	**Validation**
Ability to use → Perceived ease of use	0.415	***	Accepted
Course content and design → Perceived ease of use	0.276	***	Accepted
Instructor contribution → Perceived ease of use	0.042	0.312	Rejected
Previous experience in e-learning → Perceived ease of use	−0.037	0.295	Rejected
The quality of the e-learning system → The perceived ease of use	−0.015	0.737	Rejected
Ability to use → Perceived utility	0.095	0.063	Rejected
Course content and design → Perceived utility	0.472	***	Accepted
Instructor contribution → Perceived utility	−0.065	0.115	Rejected
Previous experience in e-learning → Perceived utility	0.038	0.285	Rejected
E-learning system quality → Perceived utility	0.011	0.807	Rejected
Perceived ease of use → Perceived utility	0.460	***	Accepted
Perceived utility → Satisfaction and personal development	0.751	***	Accepted
Perceived utility → Attitude toward the use	0.570	***	Accepted
Perceived ease of use → Satisfaction and personal development	0.167	0.025	Rejected
Perceived ease of use → Attitude toward the use	0.109	0.057	Rejected
Perceived utility → Behavioral intent to use	0.262	***	Accepted
Perceived ease of use → Behavioral intent to use	-.016	0.791	Rejected
Satisfaction and personal development → Behavioral intention to use	0.614	***	Accepted
Attitude toward use → Behavioral intention to use	0.294	***	Accepted
Behavioral intent to use → Actual use	0.142	***	Accepted
Behavioral intent to use → Academic performance	0.454	***	Accepted

*** 0.001.

As it results from validating the assumptions from the external factors, the content and design of the elective course and the use skills are validated. The use of skills by teachers is a vital percentage when it comes to e-learning. It is also imperative to think about personal wellbeing when it comes to personal skills, as it can significantly contribute to how knowledge is transferred. A vital issue during the pandemic also affected self-control; if we thought we were isolated, we would face developed IT knowledge. Acquiring technical skills and teaching skills at the same time has been a challenge for many teachers. However, it is a necessary process, due to the complexity and multifaceted nature of e-learning. The study results show that teachers do not consider having acquired practical teaching skills and methodology during the pandemic. On the other hand, this knowledge gained in the approach to e-learning has been exchanged and deepened, especially between specialist colleagues and teachers. Our results align with other field results (65–67, 79).

It becomes clear that the lecturer who teaches is the most crucial factor in learning success. We note that the teachers consider that they are ready for the e-learning system, respectively, and the organization they belong to provides them with the necessary resources to access the e-learning system. Teachers who have had little previous experience with new electronic technologies have reported more often than they have had initial difficulties, and that they are overwhelmed and stressed. As a consequence, they are particularly reluctant to get involved in new media. Teachers face the challenge of rethinking these processes and critically evaluating their practical implementation.

The perceived usefulness of the information system depends on the results produced by the information system. The introduction of digitized teaching is greatly facilitated by students convinced of the benefits. Ease of use also plays a vital role in accepting the e-learning offer. It becomes clear that competencies can only be defined if there is a target framework in the university that names the scenarios intended to be implemented.

The specific benefits of using e-learning could not consistently be implemented to the satisfaction of teachers. As a result, it is often pointed out that using the platform is not always appropriate and, in some cases, it is rejected.

This study investigated the e-learning acceptance and wellbeing of teachers, especially during COVID-19, as a substitute for the traditional form of teaching in classrooms in Romania. Although there are any studies investigating students' perceptions regarding technology acceptance in the literature (57–61, 63–73), rarely any research scrutinized the teachers' perception regarding eLearning, particularly for Romania we don't find any research that study the acceptance theology model, so we consider it interesting to investigate the reactions and wellbeing of teachers in this difficulty period in that teacher don't have the possibility to teach in classroom, the only solution was to adopt e-learning. However, the weak e-learning system in Romania is a significant obstacle to keep pace with the growing educational challenges. So it was a challenge to manage a good strategy and infrastructure to continue education activities even during the crisis and in the future to establish a good and sustainable teaching and to archive the wellbeing of the teachers and students.

When discussing higher education, e-learning is regularly seen as an engine of potential change. Teachers are a vital factor when it comes to university performance and sustainability. They can be seen as guardians; however, it depends on whether e-learning is used successfully. It is difficult to accept that the classroom is being somewhat replaced with a virtual environment, but to remain competitive, it is necessary to adapt to a hybrid version.

## Conclusions

Universities' teaching and learning processes are constantly changing due to evaluation and continuous further development. This change can be triggered by strategic decisions within a university or due to external disruption. The sudden switch to distance learning caused by COVID-19 presented teachers and learners with new challenges.

In the discussion of higher education, e-learning is regularly seen as an instrument for changes in higher education. Competence development is increasingly recognized as an essential condition for the sustainable anchoring of new learning forms and media in the university, initially referring to the teachers' knowledge, skills, and attitudes on introducing, developing, and applying innovative forms of e-learning.

In addition, competence development also includes an institutional level; it also affects the ability of an organization to provide certain services.

There is pressure on institutions to use digital media with the aim of being able to keep up nationally and internationally. As already discussed, there is a lack of strategy for sensibly designing the digitisation of teaching and integrating it into the curriculum accordingly. The university management and the departments should consider and implement these aspects. The focus is often on the necessary technology to be acquired and used.

Today, thanks to the never-ending stream of data on the Internet, more and more people can acquire knowledge, not only in western industrial nations but also in the Third World. Moreover, educational platforms such as *Wikipedia* bring concise summaries of information prepared so everyone can understand. As a result, even people who do not have the opportunity for an academic education can expand their general knowledge through established information sites on the Internet. In recent years, universities and educational institutions have also increasingly put scientific study results, academic publications, and library databases online access to the public.

Wellbeing can be defined as an individual or collective state or procedure of experiencing oneself and others and conforming to the life conditions as favorable. However, wellbeing is understood and respected differently depending on the scientific context in which it is used.

Wellbeing is sometimes also equated with positive and negative affective components, with happiness, life satisfaction, quality of life, wellness, and the negation of illness, anomie, or health.

Subjective wellbeing is the result of comparisons. These relate to judgments of the extent to which needs, standards of value, and attitudes have been adequately met. Objective wellbeing describes the living conditions necessary for the personal form. As a rule, a society's economic, ecological, and human capital is surveyed, i.e., its structural and security-providing possibilities to individually and collectively create the prerequisites for subjective wellbeing.

Digital forms of learning can also have an economic side, especially when online courses become fee-based. Nevertheless, industrial companies and representatives from universities and politics are promoting the digitisation of teaching, which often refers to further education and less to the undergraduate bachelor's degree.

The main limitation of our paper is that used data obtained by the sampling, maybe we can extend our research to greater number of respondents to have a better understanding of what is happening in the universities and how the teacher find the challenge about the e-learning and how they fell in this period of sanitary crises. Thus, we recommend more studies concerning technology acceptance for education purposes in Romania to cope with existing challenges regarding e-learning adoption.

## Data availability statement

The raw data supporting the conclusions of this article will be made available by the authors, without undue reservation.

## Author contributions

GC and MF: conceptualization. TB: methodology and funding acquisition. MF: software and data curation. XH: validation and visualization. SS: investigation. GC: resources. GC and TB: writing—original draft preparation. CI and MF: writing—review and editing and supervision. CI: project administration. All authors critically revised the manuscript and gave their final approval of the manuscript submitted for publication.

## Conflict of interest

The authors declare that the research was conducted in the absence of any commercial or financial relationships that could be construed as a potential conflict of interest.

## Publisher's note

All claims expressed in this article are solely those of the authors and do not necessarily represent those of their affiliated organizations, or those of the publisher, the editors and the reviewers. Any product that may be evaluated in this article, or claim that may be made by its manufacturer, is not guaranteed or endorsed by the publisher.
